# Hospital based palliative care in sub-Saharan Africa; a six month review from Malawi

**DOI:** 10.1186/1472-684X-10-12

**Published:** 2011-07-09

**Authors:** Julia B Tapsfield, M Jane Bates

**Affiliations:** 1 Estcourt Provincial Hospital, Old Main Road, Estcourt 3310, KwaZulu-Natal, South Africa; 2 Tiyanjane Clinic, Queen Elizabeth Central Hospital, PO Box 95, Blantyre, Malawi

## Abstract

**Background:**

The World Health Organisation recognises the importance of palliative care in an African setting. Despite this services are often patchy and inconsistent, and many operate at health centre and/or community level. Few reports from hospital based palliative care services in sub-Saharan Africa exist in the current literature. As part of its activities Tiyanjane Clinic has been providing hospital based palliative care to patients at Queen Elizabeth Central Hospital, a large government tertiary referral institution, in the Southern region of Malawi since 2003, caring for patients with HIV, cancer and other non-malignant palliative diagnoses.

**Methods:**

A retrospective review of case notes for all in-patients seen by Tiyanjane Clinic over a six month period (April-Sept 2009) was undertaken.

**Results:**

A total of 177 patients were seen, for whom 137 case notes were available (77%). 58% of patients were male, 42% female. The average age of patients was 39.1 years (range 15-92 years). 54% of patients were HIV positive, with 34% on ARV drugs at the time of care. 42% of patients had HIV related diagnoses, including AIDS defining malignancies, 48% had (non AIDS related) cancers and 9% had other palliative diagnoses. The mean age of patients with HIV related diagnoses was 34 years, for cancer patients it was 48 years. Pain was the most commonly reported symptom (74%), with 56% of patients requiring oral morphine. The mean daily dose of morphine was 30 mg/day (range 9-100 mg). 65% of patients were discharged home, 26% of patients died during admission.

**Conclusions:**

The palliative care population in this setting is relatively young, especially among patients with HIV related diagnoses. HIV and cancer are the main diagnostic groups. Pain is the most commonly reported symptom, with oral morphine frequently required. Health workers require access to and knowledge of oral morphine in order to provide appropriate assistance to patients under their care.

## Background

Palliative care improves the quality of life for patients and families who face life-threatening illnesses, from diagnosis through to end of life and bereavement [1]. In the developing world the majority of cancer patients present at an advanced stage of disease making palliative care an essential part of management [2]. Palliative care has also been shown to play a critical role in the management of other chronic conditions, including HIV and AIDS, even in places where anti-retroviral drugs (ARVs) are available [3,4]. Despite recognition from the World Health Organisation (WHO) of the importance of providing palliative care, studies show that the service provision in Africa remains patchy and inconsistent [5-7].

Tiyanjane Clinic was established in 2003 with the aim of improving the quality of life of patients at Queen Elizabeth Central Hospital (QECH), the largest government hospital in Malawi with over 1200 beds, and the surrounding communities. Malawi is one of the most resource poor countries in sub-Saharan Africa (SSA), with around one million of its population living with HIV and an estimated 40% of the population living in extreme poverty [8]. Per capita spending on health is around $64 per year [9]. These problems are exacerbated by critical human resource shortages in the health care sector [10].

Starting with a focus on providing holistic care for patients with HIV and AIDS related illnesses Tiyanjane Clinic provides palliative care services for in-patients referred from the adult medical wards at QECH. The clinic is situated within the hospital and referrals are taken from clinicians working on those wards using a structured referral form. Other aspects of the service, not reported on here, include HIV counseling and testing, out-patient clinics, community visits and a community based service at a nearby government run health centre. The Clinic team comprises three clinicians, three nurses, one HIV counselor and two support staff. Government health services, through the hospital, district health services and the local medical school, support the majority of the costs.

This review looks at six months of in-patient palliative care services at Tiyanjane Clinic.

## Methods

A retrospective paper based case-notes review of in-patients managed by Tiyanjane Clinic over a six month period (April - September 2009) was undertaken. An ethics waiver from the University of Malawi, College of Medical Research and Ethics was obtained to perform our study. Clinic records were used to generate a list of all in-patients at QECH who were seen by Tiyanjane over this time period. There were no exclusion criteria with all patients seen over this time period included in the study. Initially paper notes, the principle source of patient records, and then the clinic's computer database were searched to obtain comprehensive records for each patient. Each set of notes was used to identify data on patient age, sex, diagnosis, reported symptoms, medications prescribed, length of stay and outcome. All records were anonymous for identifying patient details. The data was recorded in and analysed using Microsoft Excel.

## Results

A total of 177 patients were seen over a six month period. Records were obtained for 137 of these patients (77%), with the remaining 40 patient's records unobtainable. Of these 137 there were 17 patients (12%) for whom only computer records were available.

**Figure 1:**
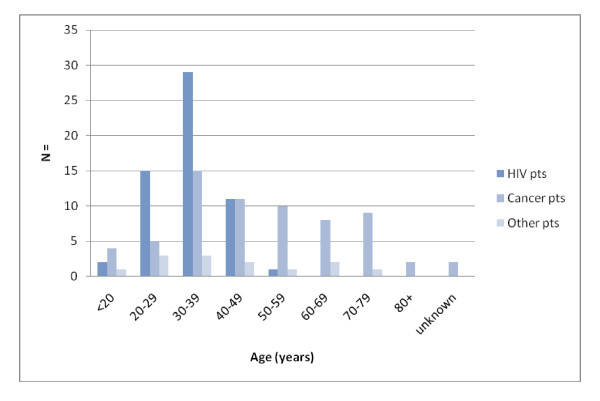
**Patient age range, by diagnostic group**.

**Figure 2:**
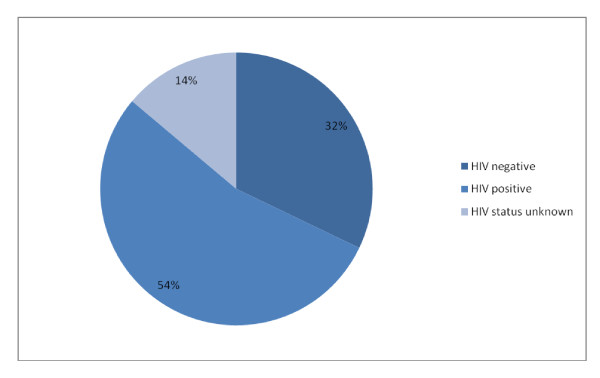
**HIV status (all patients)**.

79 patients were male (58%) and 58 were female (42%). The mean age of the patients was 39.1 years (range 15 - 92 years). 74 patients (54%) were known to be HIV positive, with 25 (34%) already on ARVs at time of care. (Table 1 and 2, Figures 1 and 2)

**Table 1:** Patient sex ratios

	All pts	*(%)*	HIV pts	*(%)*	Cancer pts	*(%)*	Other pts	*(%)*
**Sex**								

Male	79	*58*	33	*57*	40	*61*	6	*46*

Female	58	*42*	25	*43*	26	*39*	7	*54*

Total	137	*100*	58	*100*	66	*100*	13	*100*

**Table 2:** HIV status of patients

	All pts	*(%)*	HIV pts	*(%)*	Cancer pts	*(%)*	Other pts	*(%)*
Negative	44	*32*	0	*0*	37	*56*	7	*54*

Positive	74	*54*	58	*100*	13	*20*	3	*23*

Unknown	19	*14*	0	*0*	7	*23*	3	*11*

**HIV pts on ARVs?**								

On ARVs	25	*34*	38	*66*	5	*38*	3	*100*

Not on ARVs	46	*62*	17	*29*	8	*62*	0	*0*

Unknown	1	*1*	1	*2*	0	*0*	0	*0*

Stopped	2	*3*	2	*3*	0	*0*	0	*0*

**Figure 3:**
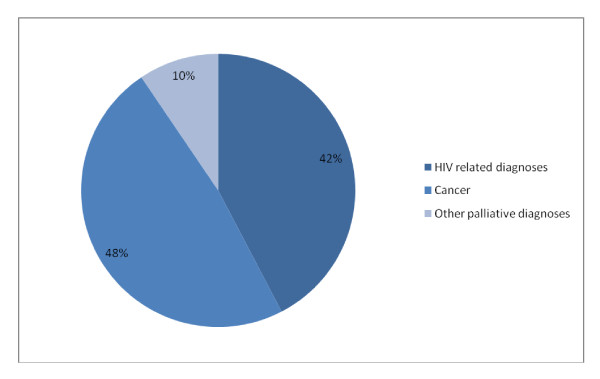
**Patient diagnoses**.

66 patients (48%) had a diagnosis of (non-AIDS related) cancer. 40 patients (29%) had an AIDS defining malignancy (as defined by the WHO clinical staging of HIV/AIDS for adults and adolescents [11]), mainly Kaposi's sarcoma (37patients). 18 (13%) had other diagnoses related to HIV, including cryptoccocal meningitis, lactic acidosis secondary to ARVs, "advanced HIV" and progressive multifocal leukoencephalopathy, giving a total of 58 patients (42%) with HIV related diagnoses. The remaining 13 patients (9%) had other palliative diagnoses, including heart failure and/or cardiomyopathy (4 patients), liver and/or renal failure (2 patients) and subarachnoid haemorrhage (2 patients). (Figure 3)

The mean age of patients with HIV related diagnoses was 34 years (range 15-53). For cancer patients the mean age was 48 years (range 15-92). (Figure 1)

**Figure 4:**
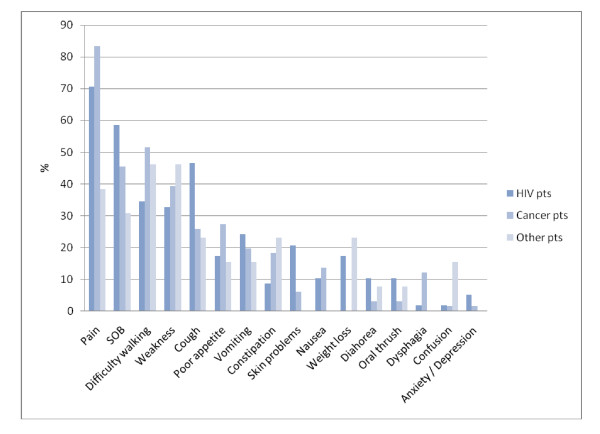
**Prevalence of reported symptoms by diagnostic group**.

Pain was the most commonly reported symptom (74%). Other common symptoms were shortness of breath (50%), being unable to walk (44%) and weakness (37%). Four patients (3%) reported symptoms of anxiety/depression. 83% of cancer patients reported pain, 71% of those with HIV related diagnoses reported pain. (Figure 4)

Analgesic drugs were prescribed to 117 of the patients (85%) during admission. 77 patients (56%) were prescribed oral morphine (liquid and/or tablets). The mean total daily dose of morphine was 30 mg (range of 9-100 mg). 64% of cancer patients were prescribed morphine; mean daily dose 34.0 mg (range 15-100 mg). 52% of patients with HIV related diagnoses were prescribed morphine, mean daily dose 25.5 mg (range 9-60 mg).

The average length of involvement by the palliative care team was 6.3 days (range 1-39 days). 89 patients (65%) were discharged home and 36 (26%) died on the ward. 33% of the patients with HIV related diagnoses died on the ward, 23% of cancer patients died on the ward. Follow-up was planned for 45 patients (51% of those discharged), with 26 of these patients actually attending. An additional 10 patients for who arranged follow-up wasn't documented were also seen again by clinic, making a total of 36 patients (40% of those discharged) followed-up after discharge. (Table 3 and 4)

**Table 3:** Patient outcomes

	All pts	*(%)*	HIV pts	*(%)*	Cancer pts	*(%)*	Other pts	*(%)*
Discharged	89	*65*	29	*50*	49	*74*	11	*85*

Died	36	*26*	19	*33*	15	*23*	2	*15*

Unknown	12	*9*	10	*17*	2	*3*	0	*0*

**Table 4:** Follow-up (FU) of discharged patients

	All pts	*(%)*	HIV pts	*(%)*	Cancer pts	*(%)*	Other pts	*(%)*
Planned FU	45	51*	18	36%*	23	47%*	4	36%*

Planned FU attended	26	58**	9	50**	15	65**	2	50**

Non-planned FU attended	10	11*	2	7*	7	14*	1	9*

Total attending FU	36	40*	11	22*	22	30*	3	27*

* % of people who were discharged

** % of people for whom FU was planned.

## Discussion

This case note review provides a good insight into a hospital based palliative care service in a low resource setting, with a high HIV prevalence, in SSA. Palliative care has recently been recognised by the Ministry of Health in Malawi as part of a minimum standard of care for all tertiary institutions (Dr Jane Mallewa, QECH, personal communication). This paper provides useful data of some of the service needs that would need to be considered when developing such a service in the same or similar settings.

The mean age of in-patients seen by the palliative care team was 39.1 years; the average life expectancy in Malawi is 53 years [12]. Other studies of palliative care populations from around the world have found a mean age of 66 years in the UK, a median of 62 years in Taiwan and a mean of 42.7 years from a hospital based palliative care service in South Africa, highlighting the lower age range which maybe expected by those providing palliative care in SSA [13-15]. The difference in age between the patients with HIV related diagnoses and cancer patients (33 years compared with 48 years) emphasises the impact of the HIV epidemic in our setting. Patients are being referred to palliative care services during what should be expected to be their most productive working years. This places a particular burden on families and has implications for patients and carers alike.

It is important to note however that more than one in five of the patients seen were either over the age of 60 (12%) or under the age of 25 (11%). Health care professionals need to be aware of the special needs and perspectives of both adolescents and young adults, and the elderly facing life threatening illnesses in this setting.

At the time of review referrals were predominately taken from general medical adult wards. More recently an outreach service to general surgical, gynaecology and other specialist wards in the hospital is being developed utilising a series of 'link nurses' based on the specialist wards who are trained to undertake palliative care assessments. There is a separate paediatric palliative care team operating at the hospital. The total number of patients requiring palliative care across all hospital specialties at our hospital during this period is likely to have been much higher.

HIV was common, with just over half of the patients HIV positive. Malawi has an HIV prevalence of 11.9%, although the urban prevalence is known to be higher at around 17% [16]. Recent studies in QECH have found an HIV prevalence of 70% among medical in-patients [17]. Only a third of the HIV positive patients were on ARVs. Reasons for patients not being on ARVs when being seen by the palliative care team may include this presentation being their first presentation of disease, and therefore the initial diagnosis of HIV, or the presence of other co-morbidities, such as TB, delaying the initiation of ARVs until a later date. ARVs have been freely available in the public sector in Malawi since 2004, with an estimated 300,000 people eligible. Currently around 200,000 are on treatment [18]. More work to improve awareness of, and access to, ARVs in Malawi is still needed to further increase the number of eligible people receiving ARVs. However it is important to emphasize that palliative care services may still be required for patients with HIV who are taking ARVs, especially for those who have treatment related side effects, poor adherence or HIV related malignancies.

There was a high prevalence of pain among patients with cancer (83%) and with HIV related diagnoses (71%). Other symptoms were also common among both sets of patients, which is consistent with previous findings that symptoms among HIV/AIDS patients are similar to those of patients with terminal cancer [19]. 56% of patient's required oral morphine. The mean daily dose of morphine prescribed by palliative care personnel in this setting, 30 mg/day, was much lower than the average dose of 100-250 mg/day found by a 2007 Cochrane review of opiate prescribing in palliative care [20]. Reasons for the lower doses are not clear and are likely to be multi-factorial. Despite the fact that it has repeatedly be shown to be safe and cost effective to use in low resource setting, with negligible rates of iatrogenic addiction, morphine is underutilised in numerous low resource settings [7,21]. Reasons for this include restrictive legislation, drug availability, lack of training, and fears over addiction and dependence [6,22].

A short mention should be made regarding symptoms of anxiety and depression. According to our results these symptoms were only reported by 4 patients. Other studies estimate that between 7% and 30% of palliative patients suffer from depression [23,24]. HIV patients in South Africa were found to exhibit high levels of psychological symptoms, with 55% of patients reporting depression and 49% anxiety [25]. There is little recognition of, and in-fact no word in Chichewa, the local language in Malawi, for depression. Whether our findings represent a true lack of symptoms or reflect under reporting in our records is not clear. Further work into evaluating psychological symptoms among our patient population using more in-depth screening tools would be helpful to address whether we are adequately screening for, and therefore responding appropriately to, these symptoms.

Hospital in-patient stays were short (mean 6.3 days). Ongoing provision of holistic care in the community is therefore vital, with family members, community home based care teams and community leaders all playing their part. Through collaboration with NGOs and community based organisations the Ministry of Health in Malawi has a clear system for training of home based care volunteers, and numerous groups operate in all regions of the country. Our programme works with over 100 community based volunteers in a nearby township area to provide ongoing care after discharge from hospital. Programmes such as the integrated community based home care (ICHC) models from South Africa [7] provide some ideas as to how such programmes may be implemented, though the dramatic paucity of resources for health care and of human resource in Malawi may limit replication.

Only 40% of patients discharged were followed up by the palliative care team. Transport has been cited by others as a major barrier in access to health services in resource poor settings [26]. As a central teaching hospital patients are often referred from distant health facilities far from the clinic. There is no government system of welfare payments to support palliative care patients in Malawi, with loss of income due to disease meaning funds for transport to attend follow-up, along with many other things, may be significantly lacking. Palliative care training at district and health centre level is vital to assist with timely and appropriate follow-up for patients in this setting.

## Limitations

This was a retrospective study. Despite careful searching 23% of data sheets (either in the computer or paper based clinic systems) were not found. Prospective data collection could have avoided this problem.

It is beyond the remit of a case note review to address the efficacy and quality of these interventions. Qualitative research concentrating on patients, and their families, experiences of the care they receive is needed to determine how care impacts upon the quality of life of users, and to help develop the service offered in response to these findings.

## Conclusions

This study provides insight into the problems faced by palliative care in- patients in a low resource, high HIV prevalence setting. Palliative care patients are younger than in settings of low HIV prevalence and many are taking ARV medications for HIV. Cancer and HIV/AIDS are the main diagnostic groups. Pain is a common symptom. Oral morphine is required frequently, but at relatively low doses, to control pain. Rates of depression are poorly recorded in routine assessments. Difficulties in following-up patients after discharge mean that outreach support and training of staff in district areas to provide palliative care is important to increase access to all patients in need of palliative care services.

## Abbreviations

HIV: Human immunodeficiency virus; AIDS: Acquired immunodeficiency disease; ARVs: Anti-retroviral therapy; WHO: World Health Organisation; QECH: Queen Elizabeth Central Hospital (Blantyre, Malawi); SSA: Sub-Saharan Africa; ICHC: Integrated community based home care.

## Competing interests

The authors declare that they have no competing interests.

## Authors' contributions

MJB came up with the idea for the study and oversaw the project and drafting of the manuscript. JBT carried out the data collection and analysis and drafted the manuscript. Both authors read and approved the final manuscript.

## Pre-publication history

The pre-publication history for this paper can be accessed here:

http://www.biomedcentral.com/1472-684X/10/12/prepub
